# Inhalation toxicity of (1→3)-β-D glucan: recent advances

**DOI:** 10.1080/09629359791587

**Published:** 1997-11

**Authors:** Birgitta Fogelmark, Margareta Sjöstrand, David Williams, Ragnar Rylander

**Affiliations:** 1 Department of Environmental Medicine University of Gothenburg Medicinaregatan 16 Gothenburg S-413 90 Sweden; 2 Department of Surgery James H. Quillen College of Medicine Johnson City Tennessee USA

## Abstract

To investigate the effects of (1→3)-β-D-glucan after inhalation, animals were exposed to different forms of glucan and the number of lung lavage cells was determined 24 h after exposure. None of the different forms assayed caused any increase in cell numbers. In animals exposed to endotoxin, all types of cells were increased after 24 h. A simultaneous exposure to curdlan reduced this increase in a dose-related fashion. The results suggest that (1→3)-β-D-glucan-related acute injury to the lung is induced by mechanisms other than those induced by inflammagenic agents such as endotoxin.


Mediators of Inflammation, 6, 263–265 (1997)

### Inhalation toxicity of (1→3)-β-D-glucan: recent advances

Birgitta Fogelmark[1,CA], Margareta Sjöstrand[1], David Williams[2] and Ragnar Rylander[1]

[1]Department of Environmental Medicine, University of Gothenburg, Medicinaregatan 16, S-413 90 Gothenburg, Sweden, and [2]Department of Surgery, James H. Quillen College of Medicine, Johnson City, Tennessee, USA

[CA]Corresponding author
Tel: +46 31 773 3621
Fax: +41 31 825004
Email: birgitta.fogelmark@envmed.gu.se

**To investigate the effects of (1→3)-β-D-glucan after inhalation, animals were exposed to different forms of glucan and the number of lung lavage cells was determined 24 h after exposure. None of the different forms assayed caused any increase in cell numbers. In animals exposed to endotoxin, all types of cells were increased after 24 h. A simultaneous exposure to curdlan reduced this increase in a dose-related fashion. The results suggest that (1→3)-β-D-glucan-related acute injury to the lung is induced by mechanisms other than those induced by inflammagenic agents such as endotoxin.**

**Keywords:** Animal exposure, (1→3)-β-D-glucan, endotoxin

### Introduction

There is accumulating evidence that airborne microbial cell-wall agents are important risk factors for the development of respiratory disease in general and in occupational environments [1]. Whereas the effects of one such agent, bacterial endotoxin, are relatively well understood [2–4] another agent, (1→3)-β-D-glucan, has not yet been widely researched for inhalation toxicity.

As (1→3)-β-D-glucans are present in many different organic dusts, the effects after inhalation are of particular interest. Acute inhalation studies on β-D-glucan have used curdlan, a non-branched water-insoluble (1→3)-β-D-glucan. In contrast to effects seen after endotoxin inhalation, exposure to cudlan does not induce a neutrophil invasion into the airways [5]. Furthermore, the endotoxin-induced neutrophil invasion was reported to be depressed in animals pre-exposed to curdlan [5].

In another experiment, animals were exposed for 5 weeks to an aerosol of curdlan, endotoxin or a combination [6]. The repeated exposure to curdlan caused no neutrophil invasion, but endotoxin induced a slight increase in the total number of inflammatory cells. When both curdlan and endotoxin were given together, a marked inflammatory response was present, both in the airways and the lung parenchyma.

It has also been shown that the endotoxin-boosted immunoglobulin G antibody response to inhaled ovalbumin is blocked by a simultaneous exposure to curdlan (R. Rylander, P.G. Holt, manuscript submitted for publication). Taken together, these data suggest that although curdlan interferes with the normal functioning of the defense system of the lung, it does not cause an acute neutrophil inflammation.

Further work has been concerned with the interference of (1→3)-β-D-glucan on the defense mechanisms of the lung, in terms of the inflammatory response elicited by endotoxin. Here we report work on suppression of the endotoxin-induced neutrophil response and on the dose of curdlan. Data on the inhalation effects of different forms of (1→3)-β-D-glucan are also presented.

### Materials and methods

We used full-grown guinea pigs in the experiments. They were kept in an exposure cage and subjected to a continuous-flow aerosol. Different forms of (1→3)-β-D-glucan and endotoxin (*Escherichia coli* 026:B6, Difco Laboratories, Detroit, Michigan, USA) were suspended in water (100 and 25 μg/ml, respectively) and placed in a Collison spray for aerosolization. The animals were exposed to (1→3)-β-D-glucan for 4 h and to endotoxin for 40 min.

After the exposure, the animals were given a lethal injection of sodium pentothal intraperitoneally and a lung lavage was performed according to a technique described previously [6]. The lavage fluid was

© 1997 Rapid Science Publishers

**Table 1.** Inflammatory cells (×10$^6$/g lung) in lung lavage 24 h after exposure to different forms of (1→3)-β-D-glucan (100 μg/ml)

| Exposure | Macrophages | Lymphocytes | Neutrophils | Eosinophils |
|---|---|---|---|---|
| Control | 2.3 (0.4) | 0.1 (0.1) | 0.09 (0.05) | 0.7 (0.3) |
| Curdlan | 2.6 (1.6) | 0.2 (0.2) | 0.05 (0.04) | 2.3 (1.2) |
| Schizophyllan | 1.7 (0.8) | 0.0 (0.0) | 0.02 (0.01) | 0.7 (0.3) |
| Pullalan | 1.6 (0.8) | 0.1 (0.1) | 0.07 (0.07) | 1.7 (1.1) |
| Particulate glucan | 2.0 (0.7) | 0.1 (0.5) | 0.07 (0.02) | 1.3 (0.7) |
| Barley glucan | 3.0 (0.9) | 0.1 (0.1) | 0.02 (0.02) | 1.7 (0.8) |
| Grifolan | 5.5 (1.8) | 0.1 (0.1) | 0.04 (0.04) | 1.9 (0.6) |
| Grifolan* | 4.1 (2.0) | 0.0 (0.0) | 0.02 (0.01) | 1.4 (0.5) |

Values are means (SD), $n$ = 5. *1000 μg/ml.

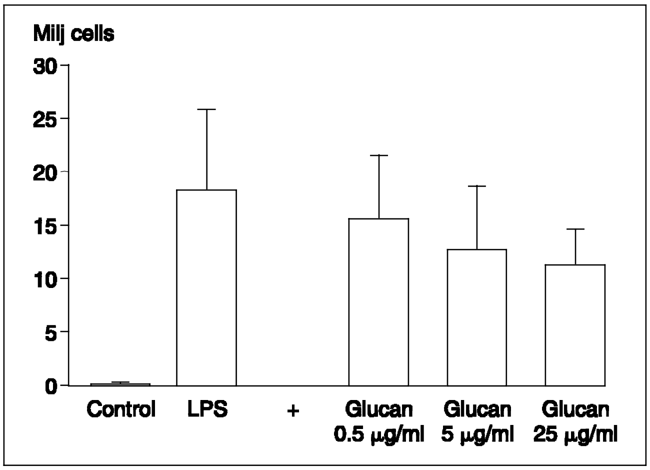

FIG. 1. Lung lavage neutrophils (×10$^6$/g lung) 24 h after 40 min of exposure to endotoxin (LPS, lipopolysaccharide) and 4 h of exposure to curdlan at different doses.

centrifuged and stained, and then a differential count of the free lung cells was performed.

### Results

Table 1 shows the cellular response after exposure to different forms of (1→3)-β-D-glucan. None elicited a change in the number of different cell types.

The endotoxin-induced neutrophil invasion into the airways after different doses of curdlan is shown in Fig. 1. Depression of the neutrophil influx after endotoxin exposure was dose-related to the amount of curdlan. A similar depression was found for eosinophils (data not shown).

### Discussion

The results from these experiments confirm earlier results indicating that curdlan does not cause neutrophilia in the airways after a single inhalation. Similar results were obtained when other forms of (1→3)-β-D-glucan were used. However, there is evidence that some forms of (1→3)-β-D-glucan may cause an inflammatory response when administered through routes other than inhalation. Sakurai *et al.* [7] injected a highly branched (1→3)-β-D-glucan isolated from *Sclerotinia sclerotiorum* intravenously in mice. Several functions of pulmonary macrophages, such as lysosomal enzyme activity and cytokine production, were elevated 1 day after the exposure and interferon-γ messenger RNA expression was elevated in lung tissue. In *in vitro* studies on murine peritoneal macrophages, Okazaki *et al.* [8] demonstrated an increase in hydrogen peroxide production after exposure to zymosan but not after exposure to gel-forming (1→3)-β-D-glucans. In a later publication [9], it was suggested that the branching ratio and molecular weight were important determinators for the induction of cytokine production. Similar findings were reported by Ohno *et al.* [10], who showed that the *in vitro* production of tumor necrosis factor-α, nitric oxide and hydrogen peroxide by mouse peritoneal macrophages was related to the single helix configuration of the (1→3)-β-D-glucan. They also showed that the (1→3)-β-D-glucan receptor binding was strongest for the triple helix configuration, which suggests that differences in the configuration of (1→3)-β-D-glucan will cause different effects. Further work is required to determine whether the depression in ovalbumin antibody formation by curdlan, which we have observed (unpublished data), is also elicited by the (1→3)-β-D-glucans that induce inflammation or whether the two processes are independent.

The present results confirm previous observations of a blocking effect on the neutrophil response after endotoxin exposure [5], and further demonstrate that this effect is dependent on the dose of curdlan. The mechanism is probably not related to the receptor function binding, as this is very specific for (1→3)-β-D-glucan. A more likely mechanism is an effect on macrophage function with a decreased secretion of chemotactic cytokines.

In summary, our results show that the effects of inhaled (1→3)-β-D-glucan are complex and that the inflammatory and immune competent cells respond differently to other agents when pretreated with glucan. This agent may therefore be an important factor in the development of pathological reactions in the lungs.

### Acknowledgements

These investigations were supported by grants from the Swedish Heart and Lung association (40027) and the Swedish Work Environment Fund (95-0287).

### References

1. Rylander R, Jacobs RR: Endotoxins in the environment: a criteria document. *Int J Occup Environ Health* 1997, **3**:S1–S48.
2. Burrell R: Immunomodulation by bacterial endotoxin. *Crit Rev Bacteriol* 1990, **17**:189–208.
3. Burrell R: Human responses to bacterial endotoxin. *Circulatory Shock* 1994, **43**:137–153.
4. Rylander R: Endotoxins in the environment. In: *Bacterial Endotoxins: Lipopolysaccharides from Genes to Therapy*. Edited by Levin J, Alving CR, Munford RS, Redl H. New York: Wiley-Liss; 1995:79–90.
5. Fogelmark B, Goto H, Yuasa K, Marchat B, Rylander R: Acute pulmonary toxicity of inhaled (1→3)-β-D-glucan and endotoxin. *Agents Actions* 1992, **35**:50–55.
6. Fogelmark B, Sjöstrand M, Rylander R: Pulmonary inflammation induced by repeated inhalations of (1→3)-β-D-glucan and endotoxin. *Int J Exp Pathol* 1994, **75**:85–90.
7. Sakurai T, Ohno N, Suzuki I, Yadomae T: Effect of soluble (1→3)-β-D-glucan obtained from *Sclerotinia sclerotiorum* on alveolar macrophage activation. *Immunopharmacology* 1995, **30**:617–622.
8. Okazaki M, Adachi Y, Ohno N, Yadomae T: Structure–activity relationship of (1→3)-β-D-glucans in the induction of cytokine production from macrophages, *in vitro*. *Biol Pharm Bull* 1995, **18**:1320–1327.
9. Okazaki M, Chiba N, Adachi Y, Ohno N, Yadomae T: Signal transduction pathway of β-glucans-triggered hydrogen peroxide production by murine peritoneal macrophages *in vitro*. *Biol Pharm Bull* 1996, **19**:18–23.
10. Ohno N, Miura NN, Chiba N, Adachi Y, Yadomae T: Comparison of the immunopharmacological activities of triple and single-helical schizophyllan in mice. *Biol Pharm Bull* 1995, **18**:1242–1247.
